# Retraction: microRNA-613 exerts anti-angiogenic effect on nasopharyngeal carcinoma cells through inactivating the AKT signaling pathway by down-regulating FN1

**DOI:** 10.1042/BSR-20182196_RET

**Published:** 2021-09-13

**Keywords:** AKT signaling pathway, FN1, Invasion, MicroRNA-613, Nasopharyngeal carcinoma

This Retraction follows an Expression of Concern and Correction relating to this article previously published by Portland Press.

This article is being retracted from *Bioscience Reports* at the request of the Editor-in-Chief and the Editorial Board following receipt of a notification from a reader, alerting the Editorial Board to duplications in the original version of Figure 3D at the time of publication, duplications in Figure 3E introduced to the paper during the Correction, and a duplicated panel in Figure 4D that overlaps with several prior publications by different authors.

The authors have been contacted in regards to the Retraction, and have not responded to the Journal's queries and the concerns raised. Given the extent of the issues raised, the Editorial Board stand by the decision to retract the article.

